# White matter disintegration in cluster headache

**DOI:** 10.1186/1129-2377-14-64

**Published:** 2013-07-24

**Authors:** Nikoletta Szabó, Zsigmond Tamás Kincses, Árpád Párdutz, Eszter Tóth, Délia Szok, Gergő Csete, László Vécsei

**Affiliations:** 1 Department of Neurology, Albert Szent-Györgyi Clinical Center, University of Szeged, Szeged, Hungary; 2 International Clinical Research Center, St. Anne's University Hospital Brno, Brno, Czech Republic; 3 Neuroscience Research Group of the Hungarian Academy of Sciences and University of Szeged, Szeged, Hungary

**Keywords:** Cluster headache, Diffusion tensor imaging, Migraine, MRI, Tract-based spatial statistics

## Abstract

**Background:**

Previous studies in primary headache disorders showed microstructural alterations in the white matter as measured by diffusion imaging. However these investigations are not in full agreement and some of those, especially in cluster headache, restricted the analysis to only a limited number of diffusion parameters. Therefore, in the current study we examined white matter microstructure in cluster headache patients.

**Methods:**

Diffusion weighted MRI images with 60 directions were acquired from thirteen patients with cluster headache and sixteen age-matched healthy controls. Tract based spatial statistics were used to compare white matter integrity in the core of the fibre bundles. Correlation of the diffusion parameters with cumulative number of headache days was examined.

**Results:**

There was a significant increment of the mean, axial and perpendicular diffusivity in widespread white matter regions in the frontal, parietal, temporal and occipital lobes. Reduced fractional anisotropy was found in the corpus callosum and some frontal and parietal white matter tracts mainly in the contralateral side of the pain. Axial diffusivity showed negative correlation to the number of the headache attacks.

**Conclusions:**

The in vivo analysis of microstructural alterations in cluster headache provides important features of the disease, which might offer a deeper insight into the pathomechanism of the disease.

## Background

Cluster headache (CH), a primary headache disorder within the group called trigeminal autonomic cephalalgias, is characterised by paroxysmal hemicrania and ipsilateral craniofacial autonomic symptoms [1]. The pathomechanism of CH is not fully understood but involves both central and peripheral mechanisms [2]. Due to the periodic appearance of the attacks, there has been numerous studies that suggest a role of the hypothalamus [3]. Positron emission tomography studies showed that the anterior cingulate cortex, the contralateral thalamus, the ipsilateral basal ganglia and both insulae were activated in CH [4,5]. Most importantly, pain related to the emotional and autonomic response is known to be the main trigger of the mentioned structures. Nevertheless, the activation of the hypothalamus seems to be a specific feature of cluster attacks [4], indicating its pivotal role in the pathogenesis and pain regulation in CH. Since the hypothalamic activation can influence the pain-matrix [6] these findings point to the multifocal origin of the CH, the dysfunction of the pain-matrix [2,7]. MRI studies found grey as well as white matter alterations in CH [8] similar to those found in migraine [9-11]. A recent diffusion tensor imaging (DTI) study in CH found reduced fractional anisotropy (FA) in the pain matrix [12]. Contrarily, another study found no microstructural alterations (investigated FA and mean diffusivity MD) in CH [8]. While these results may be contradictory, imaging markers could be a powerful tool to describe disease progression and reveal important clues on the pathomechanism. Hence, in this study we aimed to investigate the white matter microstructural alterations in CH as described with various parameters estimated from high gradient direction diffusion MRI data. In order to reduce the possible effect of the misregistration we concentrated our analysis on the core of the white matter fibre bundles as it is implemented in the Tract Based Spatial Statistics (TBSS) approach [13].

## Methods

### Participants

Thirteen patients with episodic CH and sixteen healthy controls were recruited (Table 1). The diagnosis of the CH was based on the criteria of the International Headache Society [1]. Clinical parameters such as disease duration and attack frequency were acquired for all patients. MRI scans were acquired in the interictal period. None of the subjects had white matter lesion on the conventional MRI imaging. None of the subjects suffered from depression, which was examined by Hamilton depression rating scale [14], or other neurological disorders and no one was on interval therapy. The cumulative number of headache days, referring the total number of days on which at least one cluster attack occurred, was determined based on the medical records of the patients. Control subjects did not have regular headache and did not suffer from other painful condition. The study was approved by the ethics committee of the Albert Szent-Györgyi Clinical Center (authority number: 87/2009), and all the subjects provided written consent.

**Table 1:** Demographic and clinical data of subjets

	**Patients**	**Controls**
n	13	16
Age (years)	41.1 ± 11.1	40.1 ± 8.1
Sex (male)	11	10
Disease duration (years, mean ± SD)	6.5 ± 7.1	N.A.
Right sided headache	7	N.A.
Cumulative headache days	208.2 ± 179.6	N.A
Interictal therapy	None	N.A.

Cumulative headache days are the estimated number of days spent with headace since the beginning of the disease.

### Image acquisition

The MR imaging were carried out on a 1.5 T GE Signa Excite HDxt MR scanner. 60 direction diffusion weighted images with 6 non-diffusion-weighted reference volume (TE: 93.8 ms, TR: 16000 ms, matrix: 96×96, FOV: 23×23 cm, Flip angle: 90 degree, in-plane resolution: 2.4×2.4 mm slice thickness: 2.4 mm, b: 1000 s/m2, NEX: 2, ASSET) were acquired.

### Image analysis

Diffusion data were corrected for eddy currents and movement artefacts by 12 degrees of freedom affine linear registration to the first non-diffusion-weighted reference image. Automatic extraction of the brain and cleaning from the non-brain tissues were carried out with BET [15]. Diffusion tensors at each voxel for the whole brain were fitted by algorithm, included in the FMRIB’s Diffusion Toolbox (FDT) of FMRIB’s Software Library (FSL v. 4.0, http://www.fmrib.ox.ac.uk/fsl;[16]. FA, MD, λ1 and ((λ2 + λ3)/2). Before any further processing, images were mirrored to the midsaggital axis according to the side affected by the headache. In order to reduce the possible errors arising from misalignment of images, we used the TBSS [13] method: All subjects' FA data were aligned into a common space derived from 58 high-resolution FA images of healthy subjects, using the nonlinear registration tool FNIRT. This uses a b-spline representation of the registration warp field. A mean FA image was created and set at a threshold of FA = 0.2, deriving a mean FA skeleton that represents the centres of all tracts common to the group. Each subject's aligned FA data was then projected onto this skeleton. The resulting data were fed into voxel-wise cross-subject statistics. Modelling and inference using standard general linear model (GLM) design set-up was accomplished using permutation test (5000 permutation) as implemented in FSL [17]. The design encoded for either group membership or clinical data of the patients. Statistical images were thresholded by threshold free cluster enhancing approach (TFCE) [18]. Similar analysis were carried out for the MD, perpendicular (PD) and axial diffusivity (AD). The diffusivity parameters were extracted from the regions indicated by the thresholded results of the TBSS analysis and these parameters were correlated with the number of cumulative headache days using SPSS 17. Laterality index was calculated for every diffusion parameter from the number of suprathreshold voxels [19]:

$$\mathrm{LI}=\frac{N_{L}+N_{R}}{N_{L}-N_{R}}$$

where N_L_ and N_R_ are the number of suprathreshold voxels in the left and right hemisphere.

## Results

**Figure 1:**
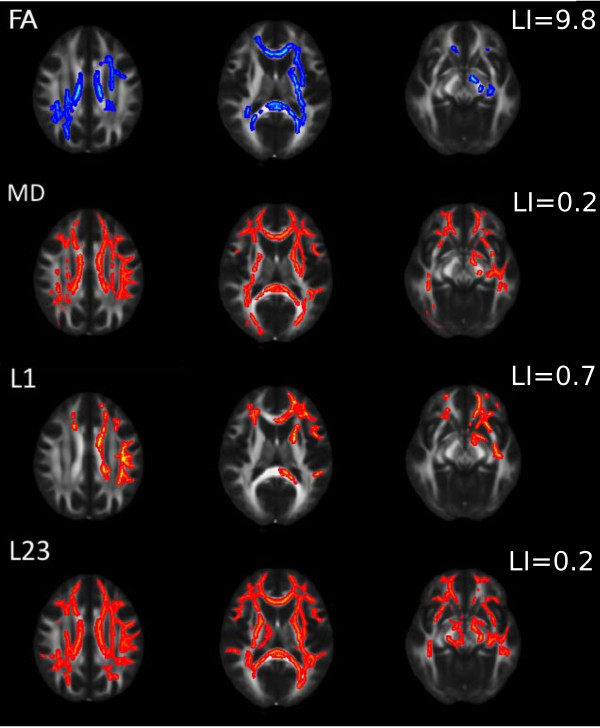
**Alteration of diffusion parameters in cluster headache patients.** Blue colours indicate reduction; red-to-yellow colours indicate increment in the given diffusion parameters. The mean FA skeleton is shown in green. A thickened version of the significant cluster is used for easier visualization (red-to-yellow or blue shades).

The whole brain TBSS analysis showed decreased FA (p < 0.02, corrected for multiple correlations) in most of the major white matter pathways: the corpus callosum, bilaterally in the forceps minor and major, right corona radiata, left internal and external capsule, left cerebral peduncule, frontal portion of the left corona radiata, right parietal juxtacortical white matter, left inferior fronto-occiptal fascicle (Figure 1, first row).

MD was found increased (p < 0.01, corrected for multiple correlations) in regions where FA alterations were found, but the alterations were more extensive involving more frontal, parietal and temporal juxtacortical white matter (Figure 1, second row).

Axial diffusivity was also found be increased in widespread white matter regions (p < 0.02, corrected for multiple correlations) similar to those of FA changes, but no significant alteration of axial diffusivity was found in the right parietal lobe in the juxtacortical white matter and the posterior corona radiate (Figure 1 third row).

**Figure 2:**
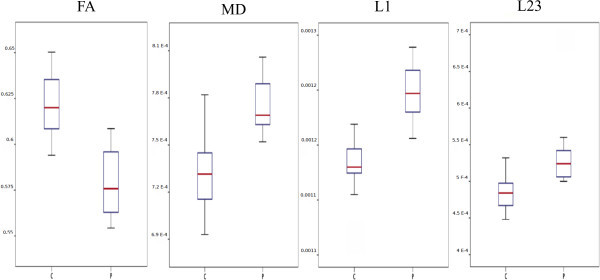
**Alteration of the diffusion parameters in the affected area.** Boxplots show the averaged parameters under the results of analyses on the white matter.

Augmented perpendicular diffusivity was the most extensive among the different diffusion parameters, involved essentially all major white matter fibre bundles, except the right external capsule (Figure 1 fourth row). Altered diffusion parameters are depicted in Figure 2.

No increased FA or decreased mean, axial, or perpendicular diffusivity was detected.

Laterality indices of all measured diffusion parameters showed left dominancy (LI_FA_: 9.8, LI_MD_: 0.2, LI_AD_: 0.7, LI_PD_: 0.2).

**Figure 3:**
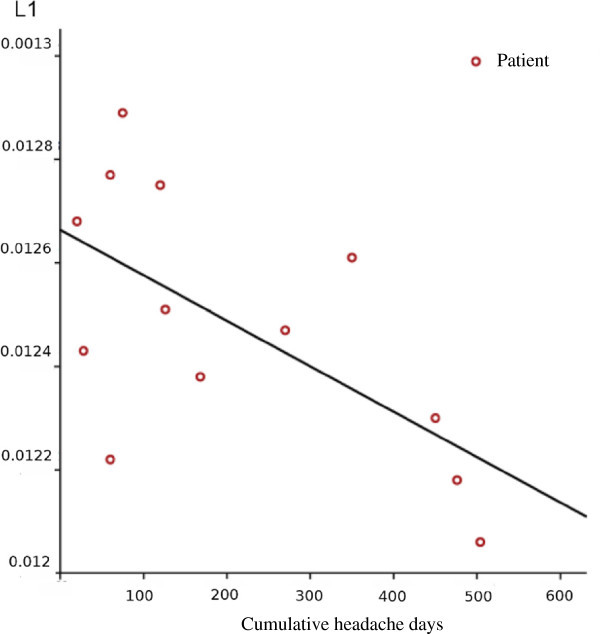
**Correlation between axial diffusivity and the number of the headache days.** Augmentation of the axial diffusivity shows inverse correlation with the cumulative headache day in lifetime.

There was a significant correlation between the number days with cumulative headache days and axial diffusivity in regions showing significant differences in AD (p < 0.022, r: 0.626, corrected for multiple comparisons, Figure 3). Other diffusion parameters did not show significant correlation.

## Discussion

In the current investigation, we found white matter microstructural alterations in cluster headache measuring diffusion in the brain with high angular resolution. The changes were widespread in the white matter but presented mainly contralateral to the side of the pain: reduced fractional anisotropy, increased mean, axial and perpendicular diffusivity in overlapping regions in the frontal, parietal, temporal and occipital lobe. Most extensive changes were detected in the mean and perpendicular diffusivity. A significant correlation was found between the cumulative headache days and axial diffusivity.

Recent investigations provided contradictory results on the diffusion alterations in cluster headache, despite using the same analysis approach as in our study. A recent DTI study described reduction of FA in several brain regions, but no other diffusion parameters were investigated [12]. The extent of the FA alterations was much smaller than in our study. Another investigation on CH patients found no alteration of FA or MD with similar analytical method [8]. One possible reason why we found more widespread changes than in previous studies could be because of the high angular resolution DTI acquisition, what we have used in our study, thereby providing a higher signal to noise ratio [20].

Correlation between the alteration of diffusion parameters and tissue microstructure is not yet entirely clear. However, the increment of mean and perpendicular diffusivity, which was the most prominent finding of our study, is most probably a sign of increased distances between membranes. This mostly relates to demyelination [21,22], but combined axon and myelin loss may also cause a complex change of diffusion parameters [23]. The increased inter-membrane distance [24] may also cause increased perpendicular diffusivity. One could speculate that the changes in the extracellular space might be related to the sterile inflammation proposed in CH [25]. However recent SPECT study did not find evidence of increased number of intracranial white blood cells in CH [26].

It was previously suggested that the lack of correlation of diffusion abnormalities with attack frequency or disease duration point to a phenotypic biomarker of the disease, reflecting a congenital condition rather than a process related to disease progression over time [8]. However in our current investigation we found a negative correlation between the axial diffusivity in and the cumulative headache days. This interesting finding can be explained by observations showing that early stages of axon damage is associated with reduced axial diffusivity [21-23,27,28]. However later, the axial diffusivity will pseudo-normalise again as the axon and myelin debris gradually cleared [29,30]. This mechanism could potentially explain our findings, nevertheless it should be emphasized that none of the DTI indices are a direct measurement of specific white matter compartments [31], hence no direct relation can be established between our results and the pathomechanism of cluster headache.

Diffusion MRI measured microstructural alterations were frequently investigated in migraine, another primary headache disorder [28-32]. In an earlier study we found similar motif of changes of various diffusion parameters to those found in cluster headache in the current study [17]. However, in migraine the extent of alterations was more focal, restricted to the frontal white matter. The similar motif of microstructural differences present in migraine and cluster headache may point to two directions: (i) Although they are quite distinct, the pathomechanism of the two diseases share some common features, [32]. (ii) Regardless of the underlying pathomechanism, migraine and cluster headache share much in the expression of pain that might be behind the diffusion alterations. In line with this hypothesis, similar diffusion changes were described in neuropathic pain in spinal cord injury [33]. Although, in two other chronic pain conditions such as irritable bowel syndrome [34] and fibromyalgia [35] increased FA was found in pain related regions with a region of interest analysis.

Functional and structural studies on cluster headache found activation and grey matter changes in the contralateral side of the pain [36-39]. Similar lateralisation of the white matter microstructural alterations were found in our investigation. Importantly, this finding point toward a mechanism different from vasodilatation of the intracranial arteries, since that is reported ipsilateral to the pain [40].

Our study is certainly not without limitation. Longitudinal studies are needed in order to reveal if the identified white matter microstructural changes are permanent. Furthermore, it would be important to know if the structural alterations have influence on brain function other than the experienced pain. Earlier studies showed that cluster headache patients have a decline of memory processing during headache attack, but not between attacks and no progressive cognitive decline was detected [41]. While our results are solely structural in nature, given the strong coupling between structure and function in the, brain functional correlates also have to be considered. Our results can be paralleled by recent experiments showing altered resting state fMRI activity in cluster headache patients [7,42]. Furthermore, investigation of the correlation between these microstructural alterations and molecular markers is imperative to get in depth understanding of the pathogenetic relevance of our findings.

## Conclusions

In conclusion, the pattern of diffusion parameter changes, what we found in cluster headache is similar to what we have previously described with identical methods in migraine [11], but the changes in cluster headache are more extensive. The identified microstructural alterations are also more extensive than it was found previously in similar studies in cluster headache [8,12], but this most probably due to methodological differences (e.g. number of diffusion directions). Our findings are important because with the detailed analysis we have used in this study, might provide possible biomarker of the disease, which could be used clinical studies.

## Competing interests

The authors declare no conflict of interest.

## Authors’ contribution

ZTK designed the study, supervised the data analysis and took part in drafting. ÁP, DS and JT selected the patients and took part in the interpretation of the results. NS participated in designing the study, carried out the MRI analysis and drafted the manuscript. ET and GC recruited the healthy subjects, carried out the MRI acquisitions, and helped in data analysis. LV took part in study design, revised the manuscript. All authors read and approved the final manuscript.
